# The first phylogenetic reconstruction of Nippostrongylinae (Nematoda: Heligmonellidae) reveals 3 new genera, the polyphyletic nature of *Carolinensis* and *Vexillata*, and identifies 5 clades with varying associations with mammals

**DOI:** 10.1017/S0031182025100395

**Published:** 2025-09

**Authors:** F. Agustín Jiménez, Guinevere O Drabik, Jorge Falcón-Ordaz, Andrew G Hope, Kurt E Galbreath, Noé U de la Sancha, John M Kinsella, Chris T McCallister, Vasyl Tkach, Whitney Preisser, Scott L Gardner

**Affiliations:** 1 School of Biological Sciences, Zoology, Southern Illinois University Carbondale, Carbondale IL, USA; 2 Laboratorio de Morfología Animal, Universidad Autónoma del Estado de Hidalgo, Pachuca, Mexico; 3 Division of Biology, Kansas State University, Manhattan, KS, USA; 4 Department of Biology, Northern Michigan University, Marquette, MI, USA; 5 Environmental Science and Studies, DePaul University, Chicago, IL, USA; 6 HelmWest, Missoula, MT, USA; 7 Division of Natural Science, Northeast Texas Community College, Mt. Pleasant, TX, USA; 8 Department of Biology, University of North Dakota, Grand Forks, ND, USA; 9 Department of Ecology, Evolution, and Organismal Biology, Kennesaw State University, Kennesaw, GA, USA; 10 The Harold W. Manter Laboratory of Parasitology, University of Nebraska, Lincoln, NE, USA

**Keywords:** comparative method, Heligmonellidae, *Lovostrongylus*, *Neoboreostrongylus*, *Tepalcuanema*

## Abstract

The Nippostrongylinae is a group of strongylid nematodes that includes species typically associated with coprophagous mammals; in the New World, it is represented by 82 species within 11 genera. Two main morphological features, the synlophe and the caudal bursa, are used to evaluate the characteristics that allow identification and classification of the organisms in the taxon. However, the analysis of these characters often requires a partial or total destruction of specimens and therefore morphological variation is studied in only a very small subset of organisms per species. To evaluate the phylogenetic signal from these characteristics, we use genetic data to reconstruct the first phylogeny for the Nippostrongylinae using nuclear and mitochondrial genes and include representatives of the most common and diverse genera. The reconstructed phylogeny features five distinct clades and allows us to identify three non-monophyletic taxa including *Carolinensis, Vexillata* and *Hassalstrongylus*. From these, *Carolinensis s. l*. is divided into four genera including *Carolinensis, Boreostrongylus, Neoboreostrongylus* n. gen. and *Tepalcuanema* n. gen. *Stunkardionema* is resurrected to include *Vexillata noviberiae* and *Hassalstrongylus* is divided into two, establishing *Lovostrongylus* n. gen. to include species that are closely related to *Guerrerostrongylus* and *Trichofreitasia*. Organisms in these three genera feature a caudal arrangement of type 2-2-1. Furthermore, species in *Hassalstrongylus sensu stricto* are more closely related to species in *Malvinema* and *Stilestrongylus*. Our results reveal the existence of an additional unnamed genus and underscore the usefulness of framing morphological characters in a comparative framework. A key for genera from the Americas is proposed.

## Graphical abstract

**Figure:**
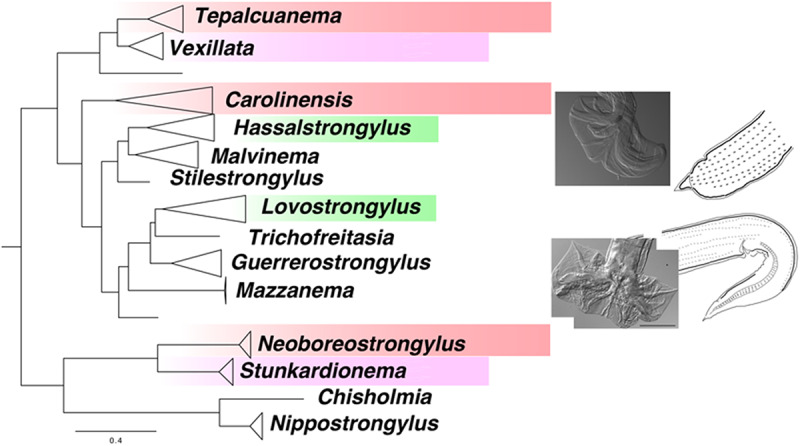

## Introduction

The Heligmosomoidea Cram, 1927, is a very diverse taxon of nematodes of tetrapods that includes species occurring mainly in rodents. They are monoxenous and upon infection via ingestion or cutaneous penetration, these nematodes feature special-level variation in their patterns of tissue migration. Because of this variation, two rodent-dwelling species, *Heligmosomoides bakeri* Durette-Desset, Kinsella and Forrester, 1972 and *Nippostrongylus brasiliensis* (Travassos, 1914), are widely used as models to study the interactions between the mammalian immune response modulation and immune evasion by the nematodes as they pass through various tissues on their way to their target site in the digestive tract (Maizels and McSorley, 2016).

The Heligmosomoidea has a complex taxonomic history in that groupings for its species diversity have been considered at different taxonomic hierarchies (Cram, 1927; Skrjabin et al., 1952; Durette-Desset and Chabaud, 1993) and used to recognize several infrafamilial taxa (Beveridge et al., 2014; Hodda, 2022). Furthermore, these monoxenous nematodes have been presumed to have a narrow host range, which has been used to justify taxonomic splitting by using the taxon of the host as a ‘character’ (Durette-Desset, 1983, 1985). However, the true degree of host range or host-specificity has been seldom tested. Most of the original descriptions offered no information relative to the simultaneous examination of additional mammals in the study site, preventing the characterization of the parasite distribution in one or in several species of sympatric mammals. Furthermore, the degree of hosts specificity has been rarely tested using molecular data.

In their natural state, as adults situated in the intestine of their host, these bursate nematodes are usually coiled, feature a cephalic vesicle and a very small buccal capsule, which in most cases is reduced to the length of a single annulus of the cuticle. These worms feature a synlophe, a system of cuticular structures that run from or near the anterior end posteriad the length of the body, these aretes, crests or cuticular ridges are typically continuous and in cross section they appear to be oriented towards the left dorsal quadrant of the body. Presently, Heligmosomoidea is recognized as a superfamily (Beveridge et al., 2014) or a subfamily Heligmosominae (Hodda, 2022) within Trichostrongylidae Leiper, 1908. The complex taxonomic history of this group of nematodes reflects numerous changes dictated by patterns of the bursal rays that are considered of taxonomic significance (Durette-Desset, 1983; Beveridge et al., 2014). The synlophe has also received attention as it is useful in the determination of major lineages within the bursate nematodes and was used to justify the proposal of infrafamilial subordinate taxa (Durette-Desset and Chabaud, 1977).

Unsurprisingly, the same set of characters is used to determine the relationships among constituent genera and species that make up the diversity of the Heligmosomoidea. Among these subordinate taxa of the Heligmosomoidea, the most diverse is the Heligmonellidae Skrjabin and Schikhobalova, 1952 which includes hundreds of species featuring an array of patterns in the caudal bursa and a spineless tail in females (Durette-Desset et al., 2017). Variation in the caudal bursa includes different patterns of branching in the dorsal ray and differences in the symmetrical arrangement of the lobes that encase the rays. Irrespective of their differences, all heligmonellids feature a buccal capsule reduced to an annulus, a cephalic vesicle and ridges in the synlophe in an oblique axis of orientation. Recent systematic efforts focused on the Heligmonellidae have evaluated cuticular and bursal structures as independent characters and provided various interpretations of their variability and usefulness as character states (Durette-Desset and Digiani, 2005a, 2012; Durette-Desset et al., 2017), but these have not been rigorously qualified through phylogenetic reconstruction that test the robustness of taxonomic classifications (de Bellocq et al., 2001). Most recently, Durette-Desset et al. (2017) recognized five subfamilies within Heligmonellidae; four of them were included in the monumental monograph of the taxon which excluded the Nippostrongylinae Durette-Desset, 1971. The Nippostrongylinae is defined by the continuous ridges along the cuticle, ridges which in cross section have a sagittal orientation: from the ventral right quadrant to the dorsal left quadrant or to the left side (Durette-Desset, 1971b, 1983). The limited set of characters available to identify more than 400 known species reduces the possible combination of characters useful for accurate diagnosis of genera and species (Durette-Desset and Digiani, 2012).

Herein we employ DNA sequence data to infer a phylogeny for species of the Nippostrongylinae present in the New World. Our objective is to establish a phylogenetic foundation for investigating morphological convergence among lineages and to identify the characters that are most informative for constructing a predictive classification. We aim to clarify classification within the most species-rich groups with emphasis on taxa representative from the Americas.

## Materials and methods

### Selection of taxa

Taxa used in this study were collected across the New World with some specimens resulting from expeditions led by the authors in both South and North America. Specific collection localities are listed in Table 1. We generated vouchers and sequences for 44 out of 47 operational taxonomic units (OTU) used in our analyses, including outgroups. Sequences of three relevant taxa that are part of the ingroup were downloaded from GenBank; these represent *Nippostrongylus brasiliensis, Nippostrongylus magnus* (Mawson, 1961) Durette-Desset, 1971 and *Chisholmia bainae* (Beveridge and Durette-Desset, 1992) Smales, 2015. We used 43 nippostrongyline worms from 10 putative genera with the goal of including at least two representative species per genus (Table 1).

**Table 1.** Specimens used in the phylogenetic reconstruction for the Nippostrongylinae of the New World, including accession numbers for GenBank and the Scientific Collections that hold the available voucher specimens. Scientific collections include Museum of Southwestern Biology, University of New Mexico (MSB: PAR); Harold W. Manter Laboratory of Parasitology, Nebraska State Museum (HWML); Colección Nacional de Helmintos, Universidad Nacional Autónoma de México (CNHE); Helminthological Collection of the Museo de La Plata, Argentina (MLP-He), and The South Australia Museum (SAM). Matrices can be located at https://doi.org/10.5061/dryad.p2ngf1w3f

TAXA	Family	Voucher	Host	Family of Host	Locality	*ITS*	*28S*	CO1	Source
*Neoboreostrongylus dalrymplei* (Dikmans, 1935) n. comb.	Heligmonellidae	MSB:PAR:24445	*Microtus pennsylvanicus* MSB:Mamm:285284	Cricetidae: Arvicolinae	14.5 km north and 32 km west of Foetus Lake, south side of Hwy 1, Northwest Territories, Canada	PV740002	PV800871	PV794349	This study
*Neoboreostrongylus dalrymplei*	Heligmonellidae	HWML:119112	*Microtus pennsylvanicus* MSB:Mamm:285642	Cricetidae: Arvicolinae	19.5 km north and 34 km west of Fort Simpson,Northwest Territories, Canada	PV739979	N/A	N/A	This study
*Neoboreostrongylus kinsellai* (Durette-Desset 1969) n. comb	Heligmonellidae	HWML:119114	*Microtus pennsylvanicus* Texas A&M University's Biodiversity Research and Teaching Collections, TCWC 66639	Cricetidae: Arvicolinae	Churchill, Manitoba, Canada	PV740003	PV800861	PV794346	This study
*Boreostrongylus minutus* (Dujardin, 1845) Durette-Desset, 1971	Heligmonellidae	N/A	-	-	Brest, France	AY332645 and AY333379	N/A	N/A	(Audebert *et al.*, 2005)
*Boreostrongylus minutus*	Heligmonellidae	N/A	*Microtus agrestis*	Cricetidae: Arvicolinae	Ceredigion, Aberystwyth, Wales, United Kingdom	ON497118	N/A	N/A	(Jackson & Friberg, 2022)
*Orientostrongylus ezoensis* (Konno, 1963) Durette-Desset, 1970	Heligmonellidae	N/A	*Rattus norvegicus*	Muridae: Murinae	Ebetu, Hokkaido, Japan	AB609319 and AB609318	N/A	N/A	(Yamada *et al.*, 2012)
*Heligmonoides speciosus* Tada, 1975	Heligmonellidae	N/A	*Apodemus argenteus*	Muridae: Murinae	Ebetu, Hokkaido, Japan	AB609321 and AB609320	N/A	N/A	(Yamada *et al.*, 2012)
*Tepalcuanema perezponcedeleoni* (Jiménez, 2012) n. comb.	Heligmonellidae	HWML:67163 SIP005	*Nyctomys sumichrasti*	Cricetidae: Tylomyinae	Los Tuxtlas, Veracruz, Mexico	JX877686	N/A	N/A	(Scheibel *et al.*, 2014)
*Tepalcuanema perezponcedeleoni*	Heligmonellidae	HWML:119115	*Nyctomys sumichrasti*	Cricetidae: Tylomyinae	Los Tuxtlas, Veracruz, Mexico	PV739982	PV800862	PV794345	This study
*Tepalcuanema perezponcedeleoni*	Heligmonellidae	HWML:119116	*Nyctomys sumichrasti*	Cricetidae: Tylomyinae	Los Tuxtlas, Veracruz, Mexico	PV739983	N/A	PV794347	This study
*Tepalcuanema perezponcedeleoni*	Heligmonellidae	With Authors	*Nyctomys sumichrasti*	Cricetidae: Tylomyinae	Los Tuxtlas, Veracruz, Mexico	PV740004	N/A	PV794356	This study
*Stunkardionema noviberiae* (Dikmans, 1935) n. comb.	Heligmonellidae	HWML:119112	*Sylvilagus floridanus*	Leporidae	Carbondale, Illinois, United States	N/A	N/A	PV794339	This study
*Stunkardionema noviberiae*	Heligmonellidae	HWML:119113	*Sylvilagus floridanus*	Leporidae	Carbondale, Illinois, United States	PV739980	N/A	PV794343	This study
*Nippostrongylus brasiliensis* (Travassos, 1914)	Heligmonellidae	N/A	*Rattus norvegicus*	Muridae: Murinae	Valencia, Spain	PP389492	N/A	N/A	(Galán-Puchades *et al.*, 2024)
*Nippostrongylus_brasiliensis*	Heligmonellidae	N/A	*Rattus rattus*	Muridae: Murinae		AY332646 and AY333380	AM039748	AP017690	(Chilton *et al.*, 2015; Yamada *et al.*, 2012)
*Nippostrongylus_magnus* (Murphy, 1961) Durette-Desset, 1971	Heligmonellidae	South Australia Museum 35791	*Rattus fuscipes*	Muridae: Murinae	Mt Disappointment, Victoria, Australia	N/A	LN715229	N/A	(Chilton *et al.*, 2015)
*Chisholmia bainae* (Gibbons and Spratt, 1995,) Durette-Desset & Digiani, 2015	Heligmonellidae	South Australia Museum 36180	*Rattus fuscipes*	Muridae: Murinae	Newlyn, Victoria, Australia	N/A	LN846131	N/A	(Chilton *et al.*, 2015)
*Lagostrongylus leporis* Fukumoto, Kamiya and Ohbayashi, 1986	Heligmonellidae	N/A	*Pentalagus furnessi*	Leporidae	Hagoshima, Amamiooshima Japan	AB610547	N/A	N/A	(Yamada *et al.*, 2012)
*Vexillata convoluta* (Caballero and Cerecero, 1943) Durette-Desset, 1972	Heligmonellidae	HWML:67172	*Cratogeomys merriani*	Geomyidae	Huitzilac, Morelos, Mexico	JX877692	N/A	JX877732	(Scheibel *et al.*, 2014)
*Vexillata convoluta*	Heligmonellidae	HWML:67172	*Cratogeomys merriani*	Geomyidae	Huitzilac, Morelos, Mexico	PV740005	N/A	JX877321	(Scheibel *et al*., 2014) This study
*Vexillata dessetae* Denke, 1977	Heligmonellidae	HWML:119117	*Heteromys* sp.	Heteromyidae	Los Tuxtlas, Veracruz, Mexico	PV739984	N/A	N/A	This study
*Vexillata armandae* Gardner, Fong, Al Banna and Raymond, 1994	Heligmonellidae	HWML:119118	*Peromyscus maniculatus*	Cricetidae: Neotominae	Riley County, Kansas, United States	PV739985	PV800863	PV794340	This study
*Carolinensis carolinensis* (Dikmans, 1935) Travassos, 1937	Heligmonellidae	HWML:119120	*Peromyscus maniculatus*	Cricetidae: Neotominae	Arkansas, United States	PV739987	PV800864	PV794336	This study
*Carolinensis neotoma* (Murphy, 1952) n. comb.	Heligmonellidae	HWML:119121	*Neotoma floridana*	Cricetidae: Neotominae	4.8 km N of Mena off St. Hwy. 88 at Blue Haze Vista, Polk County, Arkansas	PV739988	PV800865	PV794342	This study
*Carolinensis* n. sp. 1	Heligmonellidae	With Authors	*Peromyscus difficilis*	Cricetidae: Neotominae	Apaxco, Mexico	PV751009	PV800872	PV794352	This study
*Carolinensis* n. sp. 1	Heligmonellidae	HWML:119122	*Peromyscus difficilis*	Cricetidae: Neotominae	Apaxco, Mexico	PV739989	N/A	PV794348	This study
*Guerrerostrongylus marginalis* Weirich, Catzeflis and Jiménez, 2016	Heligmonellidae	With Authors	*Oecomys auyuntepui*	Cricetidae: Sigmodontinae	Cacao, French Guiana	PV740006	N/A	PV794326	This study
*Guerrerostrongylus marginalis*	Heligmonellidae	HWML:91937	*Oecomys rutilis*	Cricetidae: Sigmodontinae	Cacao, French Guiana	PV740007	PV800873	N/A	This study
*Guerrerostrongylus marginalis*	Heligmonellidae	With Authors	*Oecomys auyuntepui*	Cricetidae: Sigmodontinae	Cacao, French Guiana	PV740008	PV800874	N/A	This study
*Guerrerostrongylus zeta* (Travassos, 1937) Sutton and Durette-Desset, 1991	Heligmonellidae	MLP-He 8102	*Oligoryzomys nigripes*	Cricetidae: Sigmodontinae	**Campo San Juan, Misiones province, Argentina**	**PP447917**	N/A	N/A	(Digiani & Serrano, 2024)
*Guerrerostrongylus zeta*	Heligmonellidae	MLP-He 8103	*Oligoryzomys nigripes*	Cricetidae: Sigmodontinae	**Campo San Juan, Misiones province, Argentina**	**PP4479178**	N/A	N/A	(Digiani & Serrano, 2024)
*Guerrerostrongylus zeta*	Heligmonellidae	MLP-He 8104	*Oligoryzomys nigripes*	Cricetidae: Sigmodontinae	**Campo San Juan, Misiones province, Argentina**	**PP447919**	N/A	N/A	(Digiani & Serrano, 2024)
*Trichofreitasia* n. sp. 2	Heligmonellidae	With Authors	*Microryzomys minutus*	Cricetidae: Sigmodontinae	Yanayacu Biological Station, Cosanga, Ecuador	PV740009	N/A	PV794334	This study
*Lovostrongylus bocqueti* (Denke, 1977)	Heligmonellidae	HWML:119126	*Oryzomys*	Cricetidae: Sigmodontinae	Los Tuxtlas, Veracruz, Mexico	PV739993	N/A	PV794325	This study
*Lovostrongylus dollfusi* (Díaz-Ungría, 1963)	Heligmonellidae	HWML:119127	*Akodon montensis*	Cricetidae: Sigmodontinae	Estacion Limoy, Alto Parana, Paraguay	PV739994	N/A	PV794332	This study
*Lovostrongylus* n. sp. 3	Heligmonellidae	HWML:119123	*Zygodontomys brevicauda*	Cricetidae: Sigmodontinae	French Guiana	PV739990	N/A	PV794329	This study
*Lovostrongylus* n. sp. 3	Heligmonellidae	HWML:119124	*Zygodontomys* sp	Cricetidae: Sigmodontinae	French Guiana	PV739991	N/A	PV79433	This study
*Lovostrongylus* n. sp. 3	Heligmonellidae	HWML:119125	*Zygodontomys brevicauda*	Cricetidae: Sigmodontinae	French Guiana	PV739992	PV800866	PV794328	This study
*Lovostrongylus* n. sp. 4	Heligmonellidae	HWML 67164	*Calomys* sp.	Cricetidae: Sigmodontinae	Santa Bárbara, Jujuy, Argentina	JX877694	N/A	JX877723	This study
*Lovostrongylus* n. sp. 4	Heligmonellidae	HWML:119128	*Calomys*	Cricetidae: Sigmodontinae	Jujuy, Argentina	PV739995	N/A	PV794335	This study
*Stilestrongylus* sp. 5	Heligmonellidae	HWML:119129	*Akodon simulator*	Cricetidae: Sigmodontinae	Jujuy, Argentina	PV739996	N/A	PV794344	This study
*Stilestrongylus azarai* Durette-Desset & Sutton, 1985	Heligmonellidae	MLP-He 8101	Akodon azarae	Cricetidae: Sigmodontinae	**Berisso, Buenos Aires province, Argentina**	PP447916	N/A	N/A	(Digiani & Serrano, 2024)
*Hassalstrongylus aduncus* (Chandler 1932) Durette-Desset, 1971	Heligmonellidae	N/A	*Sigmodon hispidus*	Cricetidae: Sigmodontinae	Huehuetla, Hidalgo, México	MN044069	N/A	N/A	(Falcón-Ordaz *et al.*, 2024)
*Hassalstrongylus aduncus*	Heligmonellidae	N/A	*Sigmodon hispidus*	Cricetidae: Sigmodontinae	Huehuetla, Hidalgo, México	MN044070	N/A	N/A	(Falcón-Ordaz *et al.*, 2024)
*Hassalstrongylus aduncus*	Heligmonellidae	N/A	*Sigmodon hispidus*	Cricetidae: Sigmodontinae	Huehuetla, Hidalgo, México	MN044071	N/A	N/A	(Falcón-Ordaz *et al.*, 2024)
*Hassalstrongylus geolayarum* Falcón-Ordaz, Iturbe-Morgado, and Martínez-Salazar, 2024	Heligmonellidae	N/A	*Sigmodon hispidus*	Cricetidae: Sigmodontinae	Viborillas, Encarnacion de Diaz, Jalisco, Mexico	MN044072	N/A	N/A	(Falcón-Ordaz *et al.*, 2024)
*Hassalstrongylus geolayarum*	Heligmonellidae	N/A	*Sigmodon hispidus*	Cricetidae: Sigmodontinae	Viborillas, Encarnacion de Diaz, Jalisco, Mexico	MN044073	N/A	N/A	(Falcón-Ordaz *et al.*, 2024)
*Hassalstrongylus* sp. 6	Heligmonellidae	HWML:119130	*Sigmodon*	Cricetidae: Sigmodontinae	Los Tuxtlas, Veracruz, Mexico	PV739997	N/A	PV794333	This study
*Hassalstrongylus* n. sp. 7	Heligmonellidae	With Authors	*Microryzomysaltissimus*	Cricetidae: Sigmodontinae	Reserva ecológica El Ángel, Carchi Province, Ecuador	PV740010	PV800875	PV794327	This study
*Hassalstrongylus* n. sp. 8	Heligmonellidae	With Authors	*Microryzomys minutus*	Cricetidae: Sigmodontinae	Yanayacu Biological Station, Cosanga, Ecuador	PV740011	PV800876	PV794351	This study
*Hassalstrongylus* sp. 9	Heligmonellidae	HWML:119131	*Peromyscus* sp.	Cricetidae: Neotominae	Los Tuxtlas, Veracruz, Mexico	PV739998	PV800867 and PV800868	PV794337	This study
*Malvinema* sp. 10	Heligmonellidae	HWML:119132	*Calomys*	Cricetidae: Sigmodontinae	Jujuy, Argentina	PV739999	N/A	PV794341	This study
*Malvinema* sp. 10	Heligmonellidae	HWML:119133	*Oligoryzomys flavescens*	Cricetidae: Sigmodontinae	Jujuy, Argentina	PV740000	PV800869	PV794331	This study
*Malvinema* sp. 10	Heligmonellidae	HWML:119134	*Oligoryzomys*	Cricetidae: Sigmodontinae	Jujuy, Argentina	PV740001	PV800870	PV794330	This study
*Mazzanema* New Species 11	Heligmonellidae	With Authors	*Microryzomys minutus*	Cricetidae: Sigmodontinae	Yanayacu Biological Station, Cosanga, Ecuador	PV740012	PV800877	PV794350	This study
*Mazzanema* New Species 11	Heligmonellidae	With Authors	*Microryzomys minutus*	Cricetidae: Sigmodontinae	Yanayacu Biological Station, Cosanga, Ecuador	PV740013	PV800878	PV794354	This study
*Mazzanema* New Species 11	Heligmonellidae	With Authors	*Microryzomys minutus*	Cricetidae: Sigmodontinae	Yanayacu Biological Station, Cosanga, Ecuador	PV740014	PV800879	PV794355	This study
New Genus New Species 12	Heligmonellidae	With Authors	*Microryzomys* sp.	Cricetidae: Sigmodontinae	Reserva ecológica El Ángel, Carchi Province, Ecuador	PV740015	PV800880	PV794353	This study
*Mikenema lamothei* (Digiani, Carreño & Durette-Desset, 2008) Durette-Desset, Digiani, Kilani and Geffard-Kuriyama 2017	Heligmonellidae	CNHE 10949	*Sylvilagus floridanus*	Leporidae	Chiapas, Mexico	MN366457	N/A	N/A	(Ramírez-Cañas *et al.*, 2021)
*Ornithostrongylus quadriradiatus* (Stevenson, 1904) Travassos, 1914	Ornithostrongylidae	N/A	*Columba livia domestica*		Warsaw, Poland	OP442515	N/A	N/A	(Ledwoń *et al.*, 2023)
*Austrostrongylus victoriensis* Cassone, 1983	Herpetostrongylidae	HWML 67161	*Wallabia bicolor*	Macropodidae	Buangor, Victoria, Australia	JX877697	N/A	N/A	(Scheibel *et al.*, 2014)
*Austrostrongylus victoriensis*	Herpetostrongylidae	HWML 67162	*Wallabia bicolor*	Macropodidae	Buangor, Victoria, Australia	JX877685	N/A	N/A	(Scheibel *et al.*, 2014)
*Citellinema kinsellai* Alnaqeb, Galbreath, Koehler, Campbell and Jiménez 2022	Heligmosomidae	MSB:Para:MSB19047	*Tamiasciurus hudsonicus*	Sciuridae	South Kinaskan Lake, Stewart Cassiar Highway 37, Line 5, British Columbia, Canada	MN865429	PV800881	MN961337	(Alnaqeb *et al.*, 2022a)
*Heligmosomoides bibullosus* Alnaqeb, Greiman, Vandegrift, Campbell, Meagher, and Jiménez, 2022	Heligmosomidae	MSB:Para:MSB24583M1	*Peromyscus maniculatus*	Cricetidae: Neotominae	Klondike Highway, 22 km NW of Stewart Crossing, at mouth of Moose Creek Yukon Territory, Canada	MN865423	N/A	MN939007	(Alnaqeb *et al.*, 2022b)
*Heligmosomoides americanus* Durette-Desset, Kinsella and Forrester, 1972	Heligmosomidae	MSB:Para:MSB24546M2	*Phenacomys intermedius*	Cricetidae: Arvicolinae	Cassiar Highway (Hwy 37), Burrage River Crossing, British Columbia, Canada	MN865426	PV800882	MN927212	(Alnaqeb *et al.*, 2022b)
*Heligmosomum_mixtum* Schulz, 1954	Heligmosomidae	MSB:Para:MSB26346M1	*Myodes rutilus*	Cricetidae: Arvicolinae	40 km W Magadan, Magadan Oblast, Russia, Asia	MN865442	N/A	MN939013	(Alnaqeb *et al.*, 2022b)

### Identification of taxa

For examination, specimens were cleared in diluted glycerin and mounted on temporary slides in glycerin or glycerin jelly. For observation of the diagnostic genital structures, we dissected four male specimens to clear their posterior ends in lactophenol. Cross sections of these specimens were made to observe the synlophe at the junction of the esophagus (anterior), the midbody (mid) and in the posterior third of the worm (posterior). Preserving the last third of the body allowed us to evaluate reproductive structures of males and monodelphic prodelfic females. Based on characters observed in each individual, worms were assigned to a genus based on characteristics described in the most current diagnosis from available literature (Durette-Desset, 1970, 1983; Digiani et al., 2003, 2007; Durette-Desset and Digiani, 2005a, 2012; Durette-Desset and Guerrero, 2006; Beveridge et al., 2014).

### DNA extraction and sequencing

DNeasy Blood and Tissue spin columns (Qiagen Inc., Madison, WI, USA) were used for tissues excised between the mid-body and posterior end of the worm. The anterior and posterior ends of worms were saved as a voucher and deposited in the Harold W. Manter Laboratory of Parasitology, HWML (Lincoln, NE, USA) or the Parasite Division of the Museum of Southwestern Biology, MSB (Albuquerque, NM, USA). Attempts to extract DNA failed for specimens deposited in collections for periods longer than 15 years, including *Allipistrongylus marki* Drabik et al. (2022). One mitochondrial and two nuclear ribosomal gene regions were targeted to achieve the goals of the study. For amplification of the mitochondrial gene cytochrome c oxidase subunit 1 (*COI*), we used the primers NCOIf1 5’-CCT ACT ATG ATT GGT GGT TTT GGT AAT TG-3’ and NCO1r2 5’-GTA GCA GCA GTA AAA TAA GCA C-3’(Jiménez et al., 2013) with the following cycling conditions: 94ºC/60 s, [94ºC/10 s, 60 ºC/ 45 s, 72 º C /60 s] x 34;, 72 º C/600sec. For some reactions, we amplified COI using the universal primers LCO 5’-GGT CAA CAA ATC ATA AAG ATA TTG G-3’ and HCO 5’-TAA ACT TCA GGG TGA CCA AAA AAT CA-3’ (Folmer et al., 1994) adjusting annealing temperature to 50ºC. A continuous region of nuclear ribosomal DNA (nrDNA) including internal transcribed spacer 1 (ITS1), 5.8S and ITS2 (hereafter, *ITS*) was completed using primers NC2 and NC5 following protocols described elsewhere (Chilton et al., 2003; Jiménez et al., 2012). A second continuous region of the nrDNA including the majority of the *28S* subunit was amplified using the primers NC2R: 5’-AGC GGA GGA AAA GAA ACT AA-3’ and NC28-8 R: 5’-GTC TAA ACC CAG CTC ACG TT − 3’ with the following cycling conditions: 94°C/90 sec; [94°C/30 sec; 53°C/45 sec; 72°C/90 sec] x 34; 72⁰C/420 (Chilton et al., 2003). SydLabs HY PCR Master Mix (SydLabs, Hopkinton MA, USA) was used for all PCRs. Amplicons were submitted for Sanger sequencing at commercial facilities (MCLab, San Francisco, CA, USA; Eurofins Genomics, Louisville, KY, USA). For most products the primers used for PCR amplification were also used for sequencing. However, because of its length, *28S* was sequenced using the internal primers NC28-1, NC28R, NC28-3 NC28-12 R, NC28-5, NC28-4 R, NC28-6 R and NC28-7 described by Chilton et al. (2003). Resulting raw sequences were assembled in Sequencher version 5.4.6 (Sequencher, Ann Arbor, MI, USA) or Geneious Prime v.2020.1.2 (Biomatters, Inc., Newark, NJ, USA).

### Alignment of sequences and phylogenetic analysis

Annotated original sequences were complemented with sequences of the ingroup or relevant taxa published elsewhere and available in GenBank (Alnaqeb et al., 2022a; Alnaqeb et al., 2022b; Audebert et al., 2005; Scheibel et al., 2014; Chilton et al., 2015) The aligned mitochondrial data were analysed for the presence of pseudogenes in Mesquite v.3.5 (Maddison & Maddison., 2018), using the Muscle v.5 alignment program (Edgar, 2004). For ITS and *28S*, the alignment was performed using MAFFT software for secondary structure alignment using default QINSI settings (Katoh and Standley, 2013). The complete list of sequences generated in this study including their accession numbers are detailed in Table 1.

The models of nucleotide substitution (HKY + I + G for *28S* and GTR + I + G for *ITS* and *COI*) were selected using the best fit criteria according to the corrected Akaike Information Criterion as implemented in jModelTest v.2.1.6 (Posada, 2008). Loci were analysed phylogenetically as a concatenated dataset and the respective models of nucleotide evolution were applied to data partitions representing each locus.

The phylogenetic reconstruction of the Nippostrongylinae was performed under the optimality criteria of Maximum Likelihood using RAXML with 1,000 bootstrap replicates. Branch posterior probability was estimated using MrBayes 3.2 (Ronquist et al., 2012; Minh et al., 2020) running 4 chains for 10 million generations, with sampling every 1,000 generations and a burn-in of 25%. Convergence of the chains was assessed by examining the potential scale reduction factor and visualization of the generated TRACE plot. Analyses were completed in the CIPRES Science Gateway (Miller et al., 2010). Resulting trees were visualized using FigTree v. 1.4.4 (Rambaut, 2018).

To explore the relationships among genera revealed as paraphyletic and to expand on the taxonomic sampling density, we expanded the *ITS* dataset to include 12 additional nippostrongyline species (18 OTUs) that are only represented with this region of nrDNA in GenBank. We analysed this *ITS* datamatrix following the optimality criteria and run parameters described above. We used reciprocal monophyly and the presence of at least one synapomorphy as the criteria to designate new taxa in the genus group.

## Results

The aligned *ITS* matrix has a total length of 1,303 positions; of those positions 44% are constant and 14% are variable thus phylogenetically uninformative. The *28S* partition is 3,384 positions long, of which 38% are constant and 7% of variable positions were found to be phylogenetically uninformative. The mitochondrial *COI* loci include 677 positions: of those 34% were informative, 30% were phylogenetically uninformative and the rest were constant.

### Results of analyses of the three concatenated data partitions

**Figure 1.**
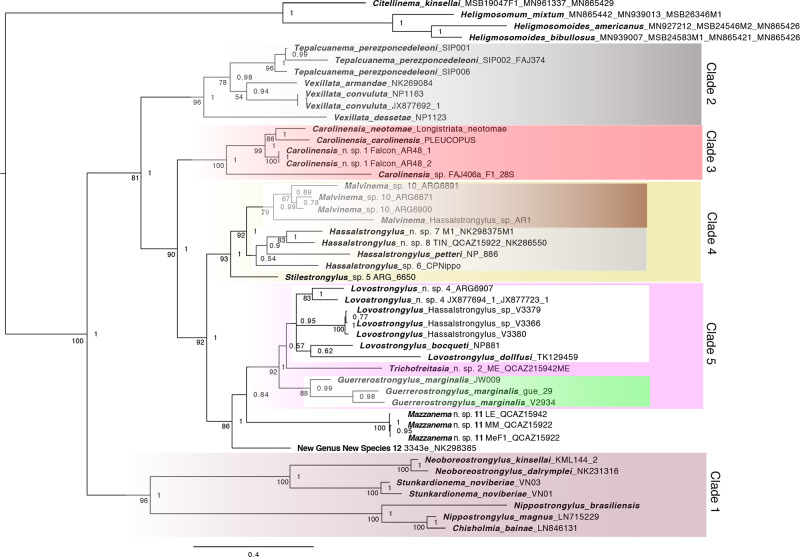
Maximum likelihood phylogeny based on concatenated nDNA (*ITS1*, 5.8, and *ITS2*) and mtDNA (*COI*) sequences. Numbers on branches indicate bootstrap support values (ML) followed by posterior probabilities (Bayesian) for major nodes. Tips are labeled with species names, followed by museum catalogue numbers and GenBank accession numbers as appropriate (Table 1).

The phylogenetic reconstruction of the concatenated dataset reveals five strongly supported clades (Figure 1). The first one, Clade 1, reveals species from the Old World, such as *Nippostrongylus brasiliensis, Nippostrongylus magnus* and *Chisholmia bainae*, as a sister group to a resolved cluster including the North American *cf. Vexillata noviberiae* (Dikmans, 1935) Durette-Desset and Digiani, 2005, *cf. Carolinensis kinsellai* (Durette-Desset 1969) Durette-Desset, 1983 and *cf. Carolinensis dalrymplei* (Dikmans, 1935) Durette-Desset, 1983. This Clade 1 is the sister group to the rest of the species in the phylogeny; from these, Clade 2 (Figure 1) includes species present in the northern Neotropics including *Vexillata armandae* Gardner, Fong, Al Banna and Raymond, 1994, *Vexillata convoluta* (Caballero and Cerecero, 1943) Durette-Desset, 1972 and *Tepalcuanema perezponcedeleoni* n. comb. (*cf. Carolinensis perezponcedeleoni* Jiménez, 2012). Clade 2 was found to be a sister group for the cluster containing the other 3 clades; among these, Clade 3 (Red in Figure 1) includes species that contain the type species for the genus *Carolinensis*, namely *Carolinensis carolinensis* (Dikmans, 1935) Travassos, 1937 in addition to *Carolinensis neotoma* (Murphy, 1952) Durette-Desset, 1983 and an unnamed *Carolinensis* sp. The rest of the taxa are contained in a clade that is further divided in two: Clade 4 (Yellow in Figure 1), which includes species of *Stilestrongylus* and *Malvinema*, and Clade 5 (Pink in Figure 1), which roughly includes species of *Hassalstrongylus, Guerrerostrongylus, Trichofreitasia, Mazzanema* and a new genus to be described separately. All resulting nomenclatural acts are sumarized in ZooBank (Table 2).

**Table 2.** Nomenclatural acts proposed based on the resulting phylogenetic reconstruction

TAXA	Authorities	Zoobank publication registration
*Neoboreostrongylus* n. gen.	Falcón-Ordaz and Jiménez, 2025	urn:lsid:zoobank.org:act:B8B34A1B-038A-4DB5-848E-60BFB0724CFA
*Neoboreostrongylus darlymplei* (Dikmans, 1935) n. comb.	This work	urn:lsid:zoobank.org:act:E145269F-102E-4776-99D1-56F43C9AFFC4
*Neoboreostrongylus kinsellai* (Durette-Desset 1969) n. comb.	This work	urn:lsid:zoobank.org:act:CE5F3DE9-8FFE-4E2F-9844-E1E2B7F3D9C9
*Neoboresotrongylus dikmansi* (Durette-Desset, 1974) n. comb.	This work	urn:lsid:zoobank.org:act:28D9A530-F87F-4CB6-8486-26C40F650EC9
*Boreostrongylus minutus*	(Dujardin, 1845) Durette-Desset, 1971	
*Tepalcuanema* n. gen.	Drabik and Jiménez, 2025	urn:lsid:zoobank.org:act:35C1ACD3-D3BC-4314-B0E1-2B1DCAEAACA1
*Tepalcuanema perezponcedeleoni* n. comb.	This work	urn:lsid:zoobank.org:act:57F011E7-3DAE-4591-8BB1-A748FB583BBF
*Stunkardionema noviberiae* n. comb.	(Dikmans, 1935) This work	urn:lsid:zoobank.org:act:25D3EF0A-45E0-4EAD-AE09-3D6955B44E30
*Lovostrongylus* n. gen	Drabik, Falcón-Ordaz and Jiménez, 2025	urn:lsid:zoobank.org:act:5660BED3-2F03-4127-8B47-407EA8650F36
*Lovostrongylus argentinus* n. comb.	(Freitas, Lent and de Almeida, 1937)	urn:lsid:zoobank.org:act:F4CFEF5E-62C4-45DC-87DE-62E6A6E707DE
*Lovostrongylus bocqueti* n. comb.	(Denke, 1977)	urn:lsid:zoobank.org:act:CD074159-70DD-4D5D-9DF2-A749FA726AC5
*Lovostrongylus dollfusi* n. comb.	(Díaz-Ungría, 1963)	urn:lsid:zoobank.org:pub:27A608EA-352E-4521-9614-74543CFA165C
*Lovostrongylus mazzai* n. comb.	(Freitas, Lent and de Almeida, 1937)	urn:lsid:zoobank.org:act:B29EA6D1-A30B-4794-A67B-DB3F76293B9D
*Lovostrongylus hoineffae* n. comb.	(Durette-Desset, 1969)	urn:lsid:zoobank.org:act:4E8D195E-0141-4BC9-A5DF-B486F1B5D4C5
*Lovostrongylus schadi* n. comb.	(Durette-Desset, 1970)	urn:lsid:zoobank.org:act:47A83722-E763-4430-AC86-3E43BF0F7F38

**Figure 2.**
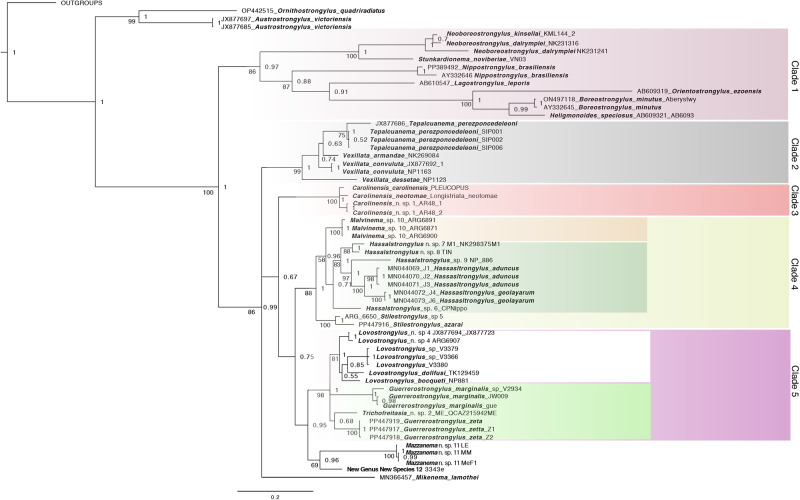
Maximum likelihood phylogeny based on the ribosomal nuclear DNA (*ITS*) sequences. Numbers on branches indicate bootstrap support values (ML) followed by posterior probabilities (Bayesian) for major nodes. Tips are labeled with species names, followed by museum catalog numbers and GenBank accession numbers as appropriate (Table 1).

The phylogeny based on *ITS* is consistent with the topology resulting from the analysis of the concatenated dataset (Figure 2). It reveals the same clades, yet lacks resolution at nodes closer to the root (*Mikenema* + *Vexillata* + *Carolinensis*). Nevertheless, the pattern reveals the same paraphyletic assemblages observed in the multi-locus phylogenetic analysis.

### Identification of non-monophyletic groups: New designation of taxa

The phylogeny shows the polyphyletic origin of taxa previously assigned as *Vexillata* Travassos, 1937. The species in Clade 1 (Maroon in Figure 2) *Stunkardionema noviberiae* (Dikmans, 1935) n. comb., was described as a species in *Longistriata* Schulz, 1926, and then transferred to *Vexillata* (*cf. Vexillata noviberiae* (Durette-Desset and Digiani, 2005b)). This same Clade 1 also contains two species formerly assigned to *Carolinensis* including *cf. Carolinensis kinsellai* and *cf. Carolinensis dalrymplei*. Their phylogenetic position warrants them to be transferred to a different taxon and morphologic similarities to species of *Carolinensis sensu stricto* should be considered to be homoplastic.

*Stunkardionema noviberiae* n. comb., is consistent with the description of the genus proposed by Arnold (1941) and very similar to the description of *Lagostrongylus* Fukumoto, Masao and Masashi, 1986 (Fukumoto et al., 1986); however the position of both species in the phylogeny (Figure 2) suggests that morphological similarities resulted from convergence. The transfer to *Stunkardionema* requires a new taxonomic act, taken herein. The other two species that act as the sister group for *Stunkardionema noviberiae* include *cf. Carolinensis kinsellai* and *cf. Carolinensis dalrymplei*; these two species were transferred to *Boreostrongylus* Durette-Desset, 1971 based on the orientation of ridges in the synlophe (Durette-Desset, 1971b). However, in the current reconstruction they do not form a monophyletic group with the type species *Boreostrongylus minutus* (Dujardin, 1845) Durette-Desset, 1971. As a consequence, we propose a new genus to include those two species.

### Neoboreostrongylus n. gen. Falcón-ordaz and Jiménez

**Figure 3.**
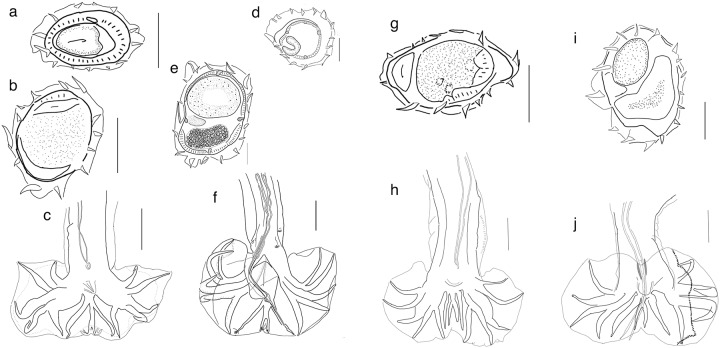
Comparison of synlophe and bursa of three genera in Nippostrongylinae. a, cross section of male and b, female of *Neoboreostronylus kinsellai*, collected from *Neofiber alleni* in Florida. c, Bursa of *Neoboresotrongylus dalrymplei* collected from *Microtus pennsylvanicus* in Canada. d, Cross section of male; e, female and f, bursa of *Tepalcuanema perezponcedeleoni* collected from *Nyctomys sumichrasti* in Los Tuxtlas locality. g, Cross section of male and h, bursa of *Carolinensis carolinensis* collected from *Peromyscus maniculatus* in Arkansas, U.S.A. i, Cross section of female and, j, bursa of *Carolinensis neotoma*, collected from *Neotoma floridana* in Arkansas, U.S.A. Scale bars **a, b, e, g,** and **h** = 30 µm. Scale bars for **c** = 200 µm. Scale bars for **d, h** and **j** = 50 µm. Scale bar for f = 100 µm

*Diagnosis*: Trichostrongylina: Heligmosomoidea: Heligmonellidae. Synlophe with 13 uninterrupted ridges. Ridges roughly oriented from right to left with dorsal ridges conspicuously smaller than the rest; sinistral (left) and dextral (right) ridges on dorsal side are relatively bigger; ventral ridges with increasing size gradient right to left (Figures 3a, b). Caudal bursa with symmetrical lobes; 2-2-1 arrangement with ray 3 longer than ray 2; ray 3 exceeds cuticular margin of bursa (Figure 3c). Dorsal ray and rays 8 share a common stalk (Figure 3c). Rays 8 split sub-symmetrically from stalk of dorsal ray at midlength; dorsal ray further divided at distal end. Genital cone prominent (>60 µm), conical in appearance and endowed with fine terminal papillae 7.

## Taxonomic summary

*Etymology*: The genus name uses the Greek prefix *Neo* to the name of the genus, in reference to their location in the New World.

*Type species: Neoboresotrongylus kinsellai* (Durette-Desset, 1969) n. comb.

*Type host: Neofiber alleni*

*Type locality:* Clewiston, Florida, U.S. A.

*Other species: Neoboresotrongylus dalrymplei* (Dikmans, 1935) n. comb.; *Neoboresotrongylus dikmansi* (Durette-Desset, 1974) n. comb.

*Other Hosts: Microtus ochrogaster*

*Other localities*: Churchill, Manitoba, Canada

### Remarks

*Neoboreostrongylus* features the typical traits of all members included in the Heligmonellidae, namely the presence of a simple buccal cavity, a synlophe made by continuous ridges with oblique axis of orientation, a monodelphic condition as well as having a simple tail without a caudal spine. The disposition and size of the ridges in the synlophe make species of this genus and *Boreostrongylus* relatively easy to differentiate because very few other taxa feature a double gradient in size of the ridges. In these two genera ridges are bigger in the flanks with smaller ridges featuring on the dorsal and ventral surfaces.

In turn, *Boresotrongylus* should include *Boreostrongylus minutus* (Dujardin, 1845), a species distributed in cricetid rodents across Eurasia (Jackson and Friberg, 2022). As a consequence, the diagnosis of *Boreostrongylus* provided by Durette-Desset (1971b) should be slightly modified to indicate that species in this genus are expected to feature 16 continuous ridges; rays 8 splitting in an asymmetrical manner from the common trunk with dorsal ray. Furthermore, the pairs of rays 7 does not feature prominently on the genital cone.

The presence of a common stalk for the dorsal ray and Rays 8 constitute a conspicuous difference in the diagnosis of *Carolinensis*. Furthermore, the synlophe in species of *Carolinensis* feature smaller ridges on the left side, and slightly larger ridges on the right side.

Relative to the phylogeny of the group, in Clade 2 (Grey Clade), there is a cluster of three species assigned to *Vexillata*, yet these are sister to a species originally assigned to *Carolinensis*, this species requires new genus that is defined below.

### Tepalcuanema n. gen. Drabik and Jiménez

*Diagnosis*: Trichostrongylina: Heligmosomoidea: Heligmonellidae. Synlophe with 13 to 16 uninterrupted ridges; oriented from dextroventral to sinistro-dorsal quadrant. Dextral lateral ridges slightly larger than others (Figures 3d, e). Caudal bursa with symmetrical lobes, pattern of type 2-2-1. Rays 2 and 3, and 5 and 6 share a stalk. Rays 8 and dorsal ray share prominent stalk. Rays 8 split symmetrically from stalk of dorsal ray at midlength; dorsal ray divided at posterior third. Genital cone prominent sub-cylindrical covered by expanded foldable cuticle (Figure 3f); basis of genital cone endowed with ventral membrane. Gubernaculum present. Females monodelphic, tail short and simple, covered by flexible cuticle that covers the tail as a sleeve.

## Taxonomic summary

*Etymology*: The genus is a combination of Nahualt and Greek. *Tepal* roughly translates into ‘from somoene’; *Cuana* translates into ‘feeding at the expense of someone’ and the Greek word *Nema* means ‘thread.’

*Type and only species: Tepalcuanema perezponcedeleoni* (Jiménez, 2012) new combination

Type locality: Adolfo López Mateos, Veracruz, Mexico

Type host: *Nyctomys sumichrasti*

### Remarks

*Tepalcuanema* is the sister taxon of *Vexillata* and yet, both genera are strikingly different because members of the latter feature a prominent *carenee*, whereas members of the former feature ridges that are roughly similar in size. *Tepalcuanema* shares several features with some species in *Carolinensis* Travassos, 1937 listed in Jiménez (2012), including the symmetrical nature of the caudal bursa and the number of ridges in the synlophe, which ranges between 13 and 16; nevertheless, while *Tepalcuanema* feature a common stalk between rays 8 and the dorsal ray, in species of *Carolinensis* both dorsal ray and rays 8 bifurcate immediately at the root (see Figures 3h, j). *Tepalcuanema* also resembles species in *Neoboreostrongylus* in that these show a prominent genital cone, ray 8 splitting at mid length of the stalk of dorsal ray, and a dorsal ray that bifurcates into rays 9 and 10 at its distal third. Furthermore, both *Neoboreostrongylus dalrymplei* and *Neoboreostrongylus kinsellai* feature a pair of subterminal papillae -papillae 7- in the genital cone, which are also present, albeit in tandem, in *Tepalcuanema perezponcedeleoni*.

Based on the continuous ridges in the synlophe and the presence of a hypertrophied genital cone, *Tepalcuanema* also exhibits similarities with some species in *Malvinema* Digiani, Sutton, and Durette-Desset, 2003, *Stilestrongylus* Freitas, Lent and Almeida, 1937, and *Suttonema* Digiani and Durette-Desset, 2003. However, males in these 3 genera show an asymmetrical caudal bursa and dorsal rays with arrangement different from 2-2-1. *Tepalcuanema* is also different from any of these genera in the relative size of the dorsal ray. The elongated dorsal ray and the symmetrical bursa of *Tepalcuanema* gives this structure the appearance of an inverted heart-shaped cup. In this regard, the bursa of *Tepalcuanema* is very similar to the homologous body parts in *Calypsostrongylus* Schmidt, Myers and Kuntz, 1967 and *Sciurodendrium* Durette-Desset, 1971. However, *Tepalcuanema* is clearly separated from them because it shows continuous ridges in the synlophe and lack of a carenee. In the hierarchical arrangement of heligmonellid nematodes, these structures are used to split the family into subfamilies (Durette-Desset, 1985). The recognition of *Boreostrongylus* and the erection of *Neoboreostrongylus* and *Tepalcuanema*, requires a redefinition of *Carolinensis*, which is represented by Clade 3 in Figure 2.

### Carolinensis Travassos, 1937

Diagnosis: Trichostrongylina: Heligmosomoidea: Heligmonellidae. Synlophe with 14 to 16 uninterrupted ridges; oriented from dextroventral to sinistro-dorsal quadrant. Ridges of different sizes; ridge 1 at sinistroventral quadrant larger than ridge 1 at dextrodorsal quadrant; ridges on dorsal side feature a decreasing size gradient from ridge 6 to 2, right to left (Figures 3g, h). Caudal bursa with subsymmetrical lobes, left lobe slightly larger, subventral rays of pattern 2-2-1 or 1-2-2. Rays 8 arising symmetrically from basis of dorsal ray; dorsal ray divided at cranial third. (Figures 3h, j). Genital cone dome shaped or blunt at its distal end. Gubernaculum present or absent. Females monodelphic, with tapering tail.

## Taxonomic summary

*Type species: Carolinensis carolinensis* (Dikmans, 1935) Travassos, 1937

*Type locality*: Great Smoky Mountains, North Carolina, U.S.A.

*Type host: Peromyscus maniculatus*

*Other species: Carolinensis norvegica* (Dikmans, 1935) Durette-Desset (1983), *Carolinensis neotoma* (Murphy, 1952); *Carolinensis peromysci* (Durette-Desset, 1974); *Carolinensis petteri* (Denke, 1977) Durette-Desset (1983), and *Carolinensis huehuetlana* Falcón-Ordaz and Sanabria-Espinoza (1996).

*Distribution*: United States of america, Mexico.

### Remarks

Travassos (1937), noted that in *Longistriata carolinensis* rays 8 and the dorsal ray bifurcated from a common root, thus there was no common trunk shared between them. He used this absence of a common trunk as the sole diagnostic character for the genus. Following, Durette-Desset (1974) transferred *Longistriata carolinensis* Dikmans, 1935, into *Boreostrongylus* Durette-Desset, 1971. As drafting the monumental taxonomic keys for the Trichostrongyloidea, Durette-Desset (1983) transferred all the species of *Boreostrongylus* into *Carolinensis*, with no justification for the combination of species featuring a prominent common trunk -*Boreostrongylus*- and those lacking a trunk -*Carolinensis*-. Subsequently, four more species were described between 1986 to 2012 including *cf. C. eothenomysi* Asakawa, Kamiya and Ohbayashi, 1986; *C. huehuetlana, cf. C. tuffi* Durette-Desset and Santos 2000, and *cf. C. perezponcedeleoni*. Based on the phylogeny presented in Figure 1, *Carolinensis* is redefined to include only species with 14 to 16 ridges with a decreasing size gradient from left to right in both ventral and dorsal sides, dome shaped genital cone, subsymmetric bursa, and rays 8 splitting from basis of dorsal ray. The gubernaculum appears to be absent in at least two species currently recognized.

The very general definition provided by Travassos (1937), combined with the lack of a formal redefinition during the last taxonomic rearrangement (Durette-Desset, 1983), resulted in *Carolinensis* becoming a hodgepodge, which is a combination of species without adequate characterization nor descriptions and with poor diagnoses. Paradoxically, relative to the copulatory bursa, the bifurcation between rays 8 relative to the stalk of the dorsal ray appears to be a reliable trait that can be used to separate members of this genus from *Boreostrongylus* and *Neoboreostrongylus* in which rays 8 and the dorsal ray share a common trunk in both *Boreostrongylus* and *Neoboreostrongylus*. It is evident that the relevance of this character as diagnostic or a strong synapomorphy was inappropriately abandoned in favor of the characters of the synlophe and caudal bursa championed during the last 40 years (Durette-Desset, 1983; Durette-Desset and Digiani, 2005a, 2012). The topology presented in the phylogenies (Figures 1 and 2) indicate that while not exclusive for this group, the proximal bifurcation between dorsal rays and ray 8 (very close to their roots) should be used in combination with the configuration of the synlophe and the ray arrangement in the lobes.

For the purposes of this comparison, *Carolinensis eothenomysi* was considered a species *inserta sedis* because neither the arrangement of ridges in the synlophe nor the presence of a common stalk supporting rays 8 and dorsal ray fit the diagnosis of the genus (Durette-Desset and Digiani, 2019). Consequently, we consider *cf. C. tuffi* also a species *inserta sedis* as it does not fit the diagnosis; even when rays 8 and dorsal ray bifurcate at their root the numbers of ridges in the synlophe is very high (20 in males, 19 in females) and the ridges do not show a clear size gradient.

Clade 4 (Yellow clade) includes *Stilestrongylus, Malvinema* and *Hassalstrongylus*; these worms feature asymmetrical bursae with an arrangement 1-4 tending to 1-3-1. In turn, Clade 5 (Pink Clade) includes members of *Trichofreitasia* and *Guerrersostrongylus*, as well as organisms that feature characters that make them fit in the definition of *Hassalstrongylus*. In their bursae, rays 3 are longer than ray 2. Because of the polyphyletic distribution of these putative *Hassalstrongylus* we propose to amend its diagnosis and propose a new genus.

### Lovostrongylus n. gen. Drabik, Falcón-Ordaz and Jiménez

**Figure 4.**
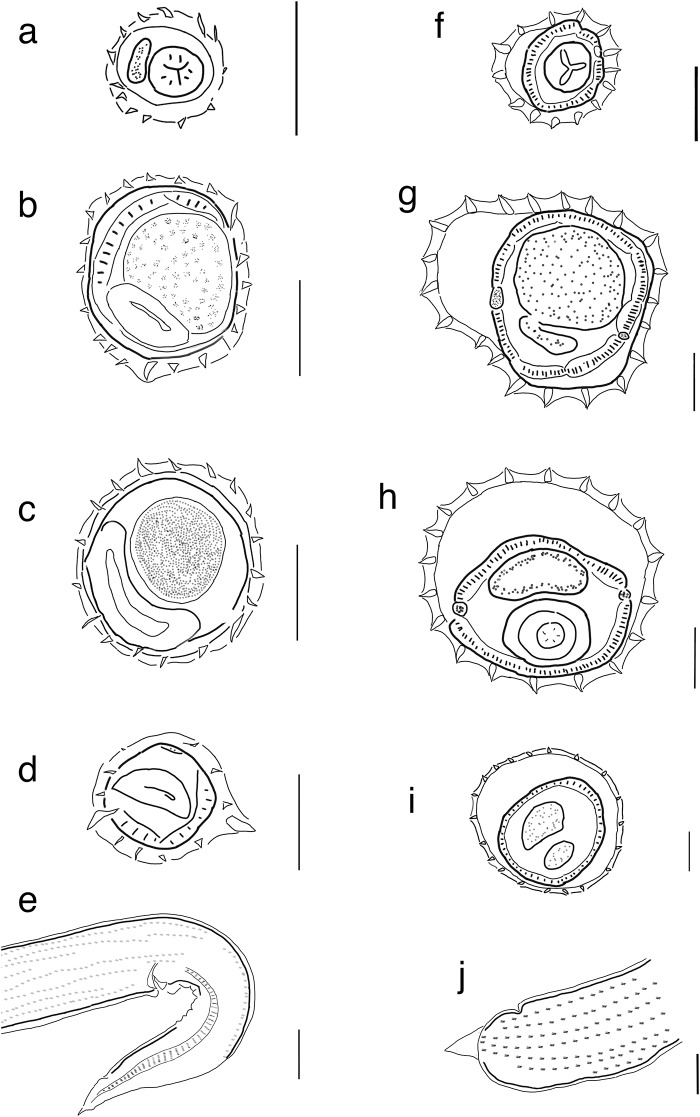
Comparison of synlophe and caudal ornamentation between *Lovostrongylus* and *Hassalstrongylus.* **a–e**, Female of *Lovostrongylus* n. sp. 4 collected from *Calomys* sp. in Argentina; a, cross section at esophageal level; b, midbody, c, uterus, d, anal region and e, posterior end in lateral view featuring postanal ala. **f–j**, female of *Hassalstrongylus geolayarum* collected from *Sigmodon* sp in Mexico; f, cross section at esophageal level, g, midbody; h, uterus, i, anal region and, j, posterior end in lateral view. Scale bars **a–d**, and **f–i** = 30 µm; **e, j**= 50 µm

*Diagnosis*: Trichostrongylina: Heligmosomoidea: Heligmonellidae. Synlophe usually with 19 to 24 uninterrupted ridges at midbody, may reach 31; oriented from sinistroventral to dextrodorsal quadrant. Ridges slightly unequal in size, with one or two prominent ridges on the dextrodorsal quadrant (Figures 4a–d). Axis of orientation of ridges from the dextroventral quadrant to the left or dorsosinistral quadrant. Caudal bursal with asymmetrical lobes; right lobe with pattern of type 2-2-1 tending to 1-3-1; left lobe with pattern of type 2-3 tending to 2-2-1. Rays 2 shorter than rays 3 and curved toward median line; rays 4 and 5 diverging at extremity. Rays 6 diverging from common trunk of rays 2-6. Rays 8 typically arising symmetrically from base of dorsal ray. Dorsal ray thickened at base, dividing within middle third into two branches; dorsal ray typically shorter than rays 8. Genital cone conical or triangular in ventral view. Gubernaculum and telamon present. Females monodelphic, with postvulvar subventral alae (Figures 4d, e); tail short, simple and protrusible as tail is covered by flexible cuticle that acts as a sleeve.

## Taxonomic summary

*Etymology*: The genus name is a combination of the Greek words lovó (‘*λοβό*’ meaning loincloth) and strongylós (‘Στρογγυλός’) meaning round. The name refers to the subventral alae around the vulva.

*Type species: Lovostrongylus argentinus* (Freitas, Lent and de Almeida, 1937) new combination

*Type host: Holochilus chacarius*

*Type locality*: Salta, Argentina

Other species: *Lovostrongylus mazzai* (Freitas, Lent and de Almeida, 1937) n. comb.; *Lovostrongylus dollfusi* (Diaz-Ungría, 1963) n. comb.; *Lovostrongylus hoineffae* (Durette-Desset, 1969) n. comb.; *Lovostrongylus schadi* (Durette-Desset, 1970) n. comb.; and *Lovostrongylus* sp. JX877694.

Distribution: Argentina, Brazil, Colombia and Venezuela.

### Remarks

Perhaps because of the lack of size gradient in the ridges in the synlophe all species listed in *Lovostrongylus* were included in *Hassalstrongylus* Durette-Desset, 1971. However, species in *Lovostrongylus* can be differentiated based on the presence of postvulvar subventral alae and the flexible caudal cuticle of females, as well as the bursal ray arrangement in males. In *Lovostrongylus*, subventral postvulvar alae feature prominently in most species and cuticular expansions in the dorsal and ventral side of the vulva make the tail appear protrusible; the bursa is asymmetrical with a typical ray arrangement 2-2-1; dorsal ray and rays 7 bifurcate at their basis. This genus is closely related to *Guerrerostrongylus* and *Trichofreitasia* (Figures 1 and 2); members of these genera also feature a caudal cuticular expansion on females, which in some cases folds into the cuticle as a sleeve, making it appear protrusible.

*Lovostrongylus* is clearly differentiated from *Guerrerostrongylus* because in the latter both dorsal ray and rays 8 share a stalk. The dorsal ray is far longer than rays 8 while ray 6 is extremely long. Furthermore, the number of ridges in *Guerrerostrongylus* exceeds 40, nearly twice as many as in most species of *Lovostrongylus,* with the exception of *Lovostrongylus dollfusi* (Serrano et al., 2021). In contrast, *Lovostrongylus* can be differentiated from *Trichofreitasia* in the nature of the bursa, which in the latter is characterized as symmetrical with hypertrophied lobes. However, in members of both genera the ray arrangement is 2-2-1 and there is a similar number of ridges in the synlophe.

### Hassalstrongylus Durette-Desset, 1971

Diagnosis: Trichostrongylina: Heligmosomoidea: Heligmonellidae. Synlophe with 19 to 25 cuticular ridges of different sizes, oriented from sinistrovental to dextrodorsal quadrant with no defined size gradient (Figures 4f–j). Asymmetrical bursa, with pattern 1-4 or 1-3-1, rays 8 split from dorsal ray at the root of their stalk, which is broad. Rays 8 usually as long as dorsal ray. Genital cone dome-shapped. Females monodelphic. Postvulvar cuticle in females is relatively simple (Figure 4i), featuring occasional dorsal expansion or ‘inflation.’

## Taxonomic summary

*Type species: Hassalstrongylus aduncus* (Chandler, 1932) Durette-Desset, 1971.

*Type host: Sigmodon hispidus*

*Type locality*: Houston, Texas, U.S.A.

Other species: *Hassalstrongylus musculi* (Dikmans, 1935) Durette-Desset, 1974; *Hassalstrongylus lichtenfelsi* Durette-Desset, 1974; *Hassalstrongylus forresteri* Durette-Desset, 1974; *Hassalstrongylus chabaudi* Diaw, 1976; *Hassalstrongylus puntanus* Digiani and Durette-Desset, 2003 and *Hassalstrongylus geolayarum* Falcón-Ordaz, Iturbide-Morgado and Martínez-Salazar, 2024.

*Distribution*: Argentina, Brazil, Ecuador, Mexico, U.S.A.

### Remarks

There are four species in *Hassalstrongylus* that are difficult to classify in the genus given the lack of material to characterize their morphological features. These include *Hassalstrongylus dessetae* Pinto, 1978, *Hassalstrongylus musculi* (Dikmans, 1935)*, Hassalstrongylus luquei* Costa, Maldonado Jr., Bóia, Lucio and Simões 2014, and *Hassalstrongylus echalieri* Diaw, 1976. From this list, both *H. luquei* and *H. echaileri* feature a caudal bursa of type 2-2-1 as that seen in species of *Lovostrongylus*; however, the female is not known for *H. luquei* and there is no conspicuous postvulvar alae in *H. echaileri.* We suspect these species could be transferred to *Lovostrongylus* once sufficient material collected to properly describe the species and the fact that at least one is morphologically similar to the undescribed species of *Lovostrongylus* included in our phylogenetic analysis (*Lovostrongylus* n. sp. 4 in Figures 1 and 2). Only the examination of the specimens will assist in their accurate determination. From this list, *Hassalstrongylus dessetae,* a species present in eastern Brazil, features 30 ridges in the synlophe, which seems consistent with the number of ridges seen in some specimens of *Lovostrongylus dollfusi* collected in Argentina (Serrano et al., 2021). Perhaps the screening of the homologous genes for these two putative species may assist in determining whether they represent a single species with ample morphological variation in the number of spines for these represent coinfection by two species. A key difference between *Hassalstrongylus* and *Lovostrongylus* is the extention and direction of Ray 3 in the bursa, in *Hassalstrongylus* this ray is typically directed from the midline towards the sides, whereas in *Lovvostrongylus* ray 3 directs anteriad, almost parallel to ray 2.

*Hassalstrongylus* appears to be closely related to *Malvinema*, which features a very prominent genital cone and asymmetrical lobes of the copulatory bursa. It is interesting to note that both *Malvinema* and *Hassalstrongylus* are related to *Stilestrongylus*. The common characteristic for these three genera includes the asymmetrical bursa and the ray arrangement that is typically 1-4.

### A new genus

The phylogeny reveals what appears to be two lineages related to the clade formed by *Lovostrongylus, Trichofreitasia*, and *Guerrerostrongylus*. This lineage includes an undescribed species of *Mazzanema* (*Mazzanema* n. sp 11) and an undescribed genus (New Genus New Species 12). The proper description of these nematodes will be provided separately.

## Discussion

### Sampling coverage for the reconstruction of the first phylogeny for the Nippostrongylinae

This is the first phylogeny of the Nippostrongylinae based on three gene regions, 1 mtDNA and 2 nrDNA, and it includes 14 of the 18 recognized genera and representatives from genera collected across the Americas spanning the Nearctic and Neotropical regions, nine taxa from Eurasia and one from Australia. Taxonomic coverage for vouchered specimens includes 28 species, of which 10 have to be formally described and named. Because of the taxonomic density in most branches the resulting phylogeny shows good resolution overall, with the exception of some internal nodes representing relationships within certain genera (Clades 4 and 5).

In addition to the specimens used to reconstruct this first phylogeny, 6 species from the Old World are represented only by sequence data of the ITS 1 and ITS 2 regions, since their 5.8S region was not made available in GenBank. These sequences were included to get a better understanding of the relationships of genera that appear to be distributed across two or more continents, yet a consequence of this inclusion is poor resolution for a few internal nodes. The missing data apparently results in long branches for nodes that are defined by strong support values and high posterior probabilities. Nevertheless, these are considered useful because they allow the clustering of closely related taxa in phylogenetically meaningful groups. Even when they do not allow the proper testing of shared ancestry they act as the foundation to identify diagnostic traits and the framework to test these relationships with additional data and OTUs. This is particularly the case for *Lagostrongylus* and *Boreostrongylus*. The clade of *Boreostrongylus minutus, Heligmonoides speciosus* and *Orientostrongylus ezoensis* features absolute support, despite all four taxonomic units missing the ribosomal gene 5.8S. Furthermore, the inclusion of these sequences allowed us to include other members of the Heligmonellidae: Nippostrongylinae in the analysis, such as *Ornithostrongylus quadriradiatus* and *Austrostrongylus victoriensis*.

The practice described above underscores the paucity of sequences available for bursate nematodes -and for parasites in general- in the universal genetic data repositories. Furthermore, the data available in GenBank is of limited usefulness for three major reasons. First, the sequences available are seldom linked to vouchered specimens that allow the verification of the parasite identity. We posit that this linkage is necessary because it affords scientists the possibility of correcting identifications, using the specimens for taxonomic decisions and linking the specimens to a geographical location that may assist in the reconstruction of their biogeographical history (De Ley et al., 2005; Jiménez et al., 2012). Second, taxonomic representation is sparse with most species of nematodes being represented by a single sequence typically generated to attempt identification. Since reconstructions of the phylogeny for the phylum were based on the phylogenetic analysis of *28S*, this marker became common (Blaxter et al., 1998; De Ley et al., 2005). As a relatively slowly evolving gene, *28S* cannot always help resolve relationships among species or closely related lineages. That task requires of more variable sites resulting from genes/regions characterized by faster rates of substitution or mutations, such as *ITS* or *COI* (Vilas et al., 2005). The problem is currently exacerbated by much of the available genetic data not conforming to a standard marker of choice which can be used to reconstruct phylogenetic relationships at supra-familial levels. This epitomizes a third major issue in that specimen materials -evidence of an infection- are often either not available or are not usable for modern applications. Parasitologists have documented the presence of parasites across centuries, and there are well curated collections that hold resources available to researchers. However, these materials are rarely preserved through methods that allows their use in perpetuity for DNA analysis. Unlike herbarium specimens, which by virtue of being dried preserve their DNA, nematodes must be frozen or fixed and preserved in ethanol. If preserved in ethanol, our experience shows that DNA will degrade over time, even if stable conditions are guaranteed. We urge parasitologists working on nematodes towards standardized workflows (Galbreath et al., 2019) and baseline sequencing, minimally to include gene regions *ITS, 28S, COI* and *16S*, which are commonly used in systematic studies and can be included in expanded reconstructions. Rapid advances in genomic sequencing will likely enable adaptive sampling of entire mitogenomes across robust sample sizes (Badger et al., 2024), where if not already attainable through collaboration should be considered now within funding initiatives.

### General patterns of geographical distribution and association with mammalian lineages

From a biogeographic perspective, the phylogeny features two very distinctive clades. The first clade contains 10 Holarctic and 1 Australian species represented by the 11 taxonomic units in Clade 1. This clade is further divided into three clusters. One cluster is formed by murine-dwelling species in the Far East of Asia and Australia (*Nippostrongylus brasiliensis, Nippostrongylus magnus* and *Chisholmia bainae*). A second cluster that includes parasites that infect arvicoline rodents (voles) across Eurasia (*Boreostrongylus minutus*) and leporids and murine rodents in eastern Asia (*Lagostrongylus lepori, Orientostrongylus ezoensis, Heligmonoides speciosus*). These two clusters appear to be reciprocally monophyletic. The third clade includes three species from the Nearctic region including *Neoboreostrongylus kinsellai* and *Neobeostrongylus dalrymplei* parasites of voles and *Stunkardionema noviberiae* which infect rabbits. The pattern appears to suggest the presence of two independent parasite lineages in the Nearctic and in the Palearctic that follow similar associations with vole and leporid hosts. Also, the branching of these lineages is congruent with the putative origin of this lineage of parasites in murine and arvicoline rodents (Durette-Desset, 1985). To clarify linkages between the Nearctic and Palearctic diversity, further sampling of species from southeast Asia and Beringia (eastern Siberia and western North America) must be included in future biogeographical analyses.

The second clade groups the species from the New World considered as the ingroup in this analysis. It includes eight lineages of which one, *Mikenema lamothei*, is a representative of a different subfamily (Heligmonellinae Skrjabin and Schikobalova, 1952). This and congeneric parasites infect leporids and feature characteristics similar to those seen in members of Nippostrongylinae, including an axis of orientation inclined at 45º to sagittal axis (‘from the ventral right quadrant to the dorsal left quadrant’) and about 14 ridges in the synlophe (Durette-Desset et al., 2017). Unfortunately, the poor representation for this taxon does not allow resolving their relationships with the rest of the lineages. The resolution of their relationships may allow scientists to establish the relationships within the entire Family (Heligmonellidae), and to select robust morphological characters that will help to stabilize the classification.

From the other seven lineages, one includes taxa that occur in both the Nearctic and the Neotropics. In particular, *Vexillata* includes parasites of pocket gophers, pocket mice and neotomine rodents, with records ranging from central USA to northern Venezuela; in large part the distribution of these parasites mirrors the distribution of pocket mice. The other lineage includes the monotypic *Tepalcuanema* (Clade 2), which are known to infect tylomine rodents in the northern Neotropics (Los Tuxtlas). Los Tuxtlas is a relevant Neotropical locality because the extensive helminthological surveys reveal the sympatry of species of both of these lineages (Denke, 1977; Jiménez, 2012).

The vast majority of species included in the other clades were recorded from cricetid rodents. Among them, *Carolinensis* is a clade that includes mainly species associated with Nearctic neotomine and sigmodontine rodents. Few records document their infection in voles and there may be at least two species that occur in the Neotropics (Falcón-Ordaz and Sanabria-Espinoza, 1996). The rest of the species included in the six remaining lineages are chiefly associated with sigmodontine rodents.

Among these, *Malvinema, Hassalstrongylus* and *Stilestrongylus* (Clade 4), appear to be essentially Neotropical. In particular most of the species in *Malvinema* are known around the tropical and subtropical regions of Argentina, whereas species of *Hassalstrongylus* range in both northern and southern hemispheres, with three species endemic in the southern Nearctic (Durette-Desset, 1974). Furthermore, two species of *Stilestrongylus* were documented in neotomine rodents in the northern Neotropics (Falcón-Ordaz and Sanabria-Espinoza, 1999).

Finally, *Lovostrongylus, Guerrerostrongylus* and *Trichofreitasia* (Clade 5) plus *Mazzanema* and a new genus yet to be named are essentially Neotropical and restricted to South American sigmodontines. The resolution of this clade may be possible with the inclusion of representatives of different lineages from Brazil.

### Convergence in both bursal arrangement and structure of synlophe

In general terms, the phylogenetic pattern underscores the homoplastic nature of the structures in the *carenee*, size of genital cone and the number of ridges, which in several cases had been used as diagnostic for genera (Durette-Desset, 1983). The phylogeny appears to offer enough resolution to support general conclusions about the diversity of the Nippostrongylinae in the New World; and represents the diversity of the parasites clustered in five clades.

The phylogenetic pattern suggests that *Vexillata* is not related to *Ornithostrongylus*. This conclusion is supported by the analysis of the *ITS* dataset alone (Figure 2), which shows that none of the species of *Vexillata*, namely *Vexillata armandae, Vexillata convoluta* and *Vexillata dessetae* share an immediate common ancestor with *Ornithostrongylus quadriradiatus*. By including a representative of the Ornithostrongylidae in this analysis, we are now able to provide an answer to the hypotheses suggested elsewhere (Guerrero, 1984; Falcón-Ordaz and Garcia-Prieto, 2004), which posited that the genus does not belong to the Ornithostrongylinae. Furthermore, our results show that *Stunkardionema noviberiae*, a species formerly included in *Vexillata* does not share a common ancestor with species in *Vexillata.* Of the species included in the analysis, *Stunkardionema noviberiae* shares some similarities with *Lagostrongylus leporis* Fukumoto et al (1986), these similarities include the structure of the *carenee* and pattern of the bursal rays (Yamaguti, 1935; Fukumoto et al., 1986). However, the topology based on *ITS*, makes it appear as if these similarities resulted from convergence. It is important to expand on the character and taxon sampling for these taxa since they may show greater taxonomic diversity across the Holarctic.

*Neoboreostrongylus dalrymplei, Neoboreostrongylus kinsellai, Boreostrongylus minutus* and *Boreostrongylus seurati* were included in *Boreostrongylus* by Durette-Desset (1971b). Subsequently these and the remaining three species making up the genus were transferred to *Carolinensis* Travassos, 1937, based on the fact that *Longistriata carolinensis* was proposed as the type species for *Carolinensis* (Travassos, 1937; Durette-Desset, 1983) and that *Carolinensis carolinensis* was inadvertently included in *Boreostrongylus* in the proposal of the latter genus (Durette-Desset, 1974). The present phylogenetic reconstruction shows that *Neoboreostrongylus dalrymplei, Neoboreostrongylus kinsellai* and *Boreostrongylus minutus* do not share a common ancestor with *Carolinensis carolinensis*; further, support for *Neoboreostrongylus dalrymplei* and *Neoboreostrongylus kinsellai* is absolute (100%/1), yet the clustering of these two species with *Boreostrongylus minutus* is not supported based on the analysis of the *ITS* phylogeny alone. These three species are arvicoline-dwelling nematodes and they feature characters that are very similar to those present in the genus *Carolinensis*. The phylogeny underscores that those similarities are the result of convergence and highlight the relevance of the shared origin for rays 8 and dorsal ray.

The phylogeny also reveals *Carolinensis sensu lato* Durette-Desset (1983) as polyphyletic because *Longistriata carolinensis* Dikmans, 1935 (type for *Carolinensis*), *Strongylus minutus* Dujardin, 1845, (Type for *Boreostrongylus*), and *cf. Carolinensis perezponcedeleoni* do not share a common ancestor. *Carolinensis sensu stricto* must be restricted to *Carolinensis carolinensis, Carolinensis neotoma*, and two undescribed species of *Carolinensis* collected in Mexico and Illinois. This clade appears to act as the sister group to the clade that includes all the diversity of species present in the Neotropics. The species included in this analysis show a similar number of ridges making up the synlophe (between 15 and 16) and feature rays 8 that do not reach the margin of the bursa and a prominent, yet not hypertrophied genital cone.

The relative position of *Vexillata dessettae* makes the genus paraphyletic. Although the support for the clade is strong, the analysis of *ITS* sequences shows a polytomy, which suggests that additional taxa and genetic markers may be required to resolve relationships within this clade. Alternatively, the inclusion of the *28S* gene for *Vexillata dessettae* may help resolving these relationships since this conservative gene may feature greater similarity with the other two species of the genus included in the analysis. We opted to establish a new genus in this clade because the morphology of species included in the clade is so strikingly different from typical characters used to define *Vexillata*. As a consequence we propose *Tepalcuanema* as a new genus to include *Tepalcuanema perezponcedeleoni* (Jiménez, 2012) Drabik and Jiménez, 2025. We predict that increasing the taxon and character sampling from members of this clade will help resolve the genus as monophyletic.

Since its inception *Hassalstrongylus* included species occurring in sigmodontine rodents across North and South America (*i.e., Hassalstrongylus aduncus; cf. Hassalstrongylus argentinus*) featuring a relatively simple synlophe with no clear size gradient in their ridges. However, species in the genus have disparate morphological traits in the female tail and the arrangement of the bursal rays (Durette-Desset, 1971b, 1983). The phylogeny reveals that species formerly assigned to *Hassalstrongylus* represent two distant clades.

The clade that includes *Hassalstrongylus aduncus,* the type species for the genus, is closely related to *Stilestrongylus* and *Malvinema*. This clade includes several species across North America, chiefly as part of *Hassalstrongylus*, and feature a bursal ray arrangement of type 1-4 and rays 8 splitting from the dorsal ray at their root. Their asymmetrical bursa appears to be a shared character with *Stilestrongylus* and *Malvinema*, in which the asymmetry of the bursa is markedly different. This clade features very strong support. In particular, we note that the morphological similar *Malvinema* and *Stilestrongylus* are not reciprocally monophyletic, even when both of them include species that can be assigned to this genus by the asymmetrical nature of the bursa, rays 8 and elongated genital cone. Rather, *Malvinema* includes taxonomic units that act as the sister group for species on *Hassalstrongylus*.

The species included in the rest of the clades feature subventral postvulvar alae or rays 8 and dorsal ray that also split from their root, and a constant bursal ray arrangement of type 2-2-1. From these structures, the 2-2-1 arrangement is a trait shared with members of *Guerrerostrongylus* and *Tricofreitasia*. Nevertheless, the dorsal ray and ray 8 in species of the latter two genera feature a relatively prominent common stalk. In this clade, an interesting problem arises in the evaluation of the phylogeny based on *ITS*, namely the lack of resolution to separate *Trichofreitasia* sp., *Guerrerostrongylus zeta* and *Guerrerostrongylus marginalis*. Rather than suggesting the splitting of *Guerrerostrongylus*, we apply the conservative approach to retain the name until further evidence in the form of additional characters and samples are included to test their relationships.

### Identification of structures suggestive of ‘parental care’

The females of *Lovostrongylus* and *Guerrerostrongylus* feature interesting modifications in the tail, which confer them the ability to fold the cuticle to cover the vulva. These structures were illustrated in detail for *Lovostrongylus dollfusi* by Serrano et al (2021) and made evident in *Guerrerostrongylus zeta* and in *Guerrerostrongylus marginalis* by others (Weirich et al., 2016; Digiani and Serrano, 2024). In particular, the presence of subventral alae in females of *Lovostrongylus* suggests that these structures may be used in the retention of eggs upon oviposition. In this genus, the character is linked to a small number of eggs maturing in the uterus, which contrasts with the relatively high fecundity seen in most of the species of trichostrongylids. Although these cuticular structures are not unique to *Lovostrongylus* -they are also present in females of *Mikenema*- their presence in combination with an apparent low fecundity raises the question if these worms feature a form of parental care. Furthermore, these subventral alae and the cuticular fold are not the only structures that may be involved in the manipulation of eggs among the Neotropical Nippostrongylinae, since females of the three known species of *Alippistrongylus* feature an expansion that may retain eggs or assist in attachment to the small intestine (Digiani and Kinsella, 2014; Drabik et al., 2022; Lemes et al., 2024).

The inclusion of representatives of *Alippistrongylus* in the analysis may help test this hypothesis, but most importantly, they may assist in a more robust reconstruction for the genus and a better understanding of the apparent diversity of body forms that is present across South American Nippostrongylinae. Considering the hypothesized origin of the Nippostrongylinae, which posits that the lineage spread from the Palearctic into the Nearctic in the lower and middle Pliocene and then into the Neotropics in the upper Pliocene (Durette-Desset, 1971a, 1985), it seems counterintuitive that the greater diversity of body forms and genera is present across the Neotropics, rather than in the Nearctic.

### Key to genera of nippostrongylinae occurring in coprophagous mammals, chiefly cricetids in the new world

Common characteristics of these nematodes include the presence of a cephalic vesicle with buccal capsule reduced to an annulus; cuticular ridges along the body form a synlophe, typically with a sagittal axis of orientation directed from right to left. Subsymmetrical or asymmetrical bursa endowed with a genital cone, paired spicules and gubernaculum. Monodelphic females with postanal end conical in shape.

1 Tail endowed with caudal appendage…………………….. *Alippistrongylus*

1’ Tail with no appendage …………………………….…………. 2

2 Synlophe inconspicuous at midbody, if present ridges barely emergent …....................... *Hypocristata*

2’ Synlophe conspicuous…………………………………………. 3

3 Carene present ………………………………………………… 4

3’ Carene absent ………………………………………………… 6

4 Rays arranged 1-3-1. Ray 8 and dorsal with no common stalk ……....................................…. *Mazzanema*

4’ Rays arranged 2-2-1. Ray 8 and dorsal ray share common stalk ………............................…. 5

5 Ray 3 directed anteriad, emerges from margin of bursa. Females with prominent lateral ridges posterior to anus. Parasites of leporids ………………….…….……… *Stunkardionema*

5’ Ray 3 directed anteriad, does not emerge from margin of bursa. Cuticle of tail in females with ridges of uniform size. Parasites of Heteromyids and geomyids …………. *Vexillata*

6 Bursa asymmetrical in size: one lobe more prominent ………7

6’ Bursa Subsymmetrical: both lobes similar in size and shape ….….................................................……. 11

7 Different ray arrangement in right and left lobes of bursa ….…………....................................................... 8

7’ Ray arrangement in both lobes of bursa are the same, typically 4-1 ……........................................... 10

8 Ray arrangement right lobe 1-3-1 tending to 4-1; 3-1-1 for left lobe. Fourteen ridges in synlophe at midbody. Parasites of invasive muroids………………..............…………*Nippostrongylus*

8’ Ray arrangement right lobe 2-2-1 tending to 1-3-1; 2-3 for left lobe tending to 2-2-1 ………………………………………………………...............................................…………………………… 9

9 Genital cone hypertrophied: length is at least half the length of caudal bursa; rays 8 with asymmetrical branching from stem of dorsal ray ………………….....……………. *Stilestrongylus*

9’ Genital cone triangular in ventral view, rays 8 branch symmetrically from dorsal ray; females feature sublateral ad anal alae ………………….....…………….………… *Lovostrongylus*

10 Hypertrophied right lobe, synlophe with 9 ridges at midbody ….…....................................…. *Suttonema*

10’ Synlophe with 17 to 24 ridges at midbody ….…………................................................……….……. *Malvinema*

11 Ray arrangement 1-4……………………………………. 12

11’ Ray arrangement 1-3-1, rarely 2-2-1 ..…………………. 13

12 Synlophe with 14 ridges, adanal ridges form alae. Parasites of leporids ..........................…. *Mikenema*

12’ Synlophe with 19 to 25 ridges, uniform ridges reach tail of females …................................ *Hassalstrongylus*

13 Synlophe at midbody between 13 to 16 ridges ………………. 14

13’ Synlophe at midbody with more than 20 ridges ……….……. 16

14 Ray 8 bifurcates immediately at root of dorsal ray …………. *Carolinensis*

14’ Ray 8 and dorsal ray share stalk, ray 8 bifurcates at least in distal third ……....................................... 15

15 Genital cone hypertrophied: more than half of length of bursa……….........................……*Tepalcuanema*

15’ Genital cone less than half length of bursa, ray 3 emerges from bursa ……………………………………………………… *Neoboreostrongylus*

16 Synlophe at midbody with more than 30 ridges, bursa type 1-3-1 …. *Guerrerostrongylus*

16’ Synlophe at midbody with 20 ridges, bursa arrangement 2-2-1 …..................…. *Trichofreitasia*

## Data Availability

DNA alignments are available at DOI: 10.5061/dryad.p2ngf1w3f
